# Mapping the landscape of climate engineering

**DOI:** 10.1098/rsta.2014.0065

**Published:** 2014-12-28

**Authors:** P. Oldham, B. Szerszynski, J. Stilgoe, C. Brown, B. Eacott, A. Yuille

**Affiliations:** 1 One World Analytics, 3B Waterview, White Cross, Lancaster, UK; 2 Centre for the Study of Environmental Change, Department of Sociology, Lancaster University, Lancaster LA1 4YT, UK; 3 Department of Science and Technology Studies, University College London, Gower St., London WC1E 6BT, UK

**Keywords:** geoengineering, climate engineering, bibliometrics, patents, intellectual property, anticipatory governance

## Abstract

In the absence of a governance framework for climate engineering technologies such as solar radiation management (SRM), the practices of scientific research and intellectual property acquisition can de facto shape the development of the field. It is therefore important to make visible emerging patterns of research and patenting, which we suggest can effectively be done using bibliometric methods. We explore the challenges in defining the boundary of climate engineering, and set out the research strategy taken in this study. A dataset of 825 scientific publications on climate engineering between 1971 and 2013 was identified, including 193 on SRM; these are analysed in terms of trends, institutions, authors and funders. For our patent dataset, we identified 143 first filings directly or indirectly related to climate engineering technologies—of which 28 were related to SRM technologies—linked to 910 family members. We analyse the main patterns discerned in patent trends, applicants and inventors. We compare our own findings with those of an earlier bibliometric study of climate engineering, and show how our method is consistent with the need for transparency and repeatability, and the need to adjust the method as the field develops. We conclude that bibliometric monitoring techniques can play an important role in the anticipatory governance of climate engineering.

## 1. Introduction

The last decade has seen a dramatic rise in interest in the idea of climate engineering, also called geoengineering, usually defined as the large-scale, intentional manipulation of the Earth's climate system to counteract anthropogenic climate change. Important milestones include Paul Crutzen's publication in 2006 [1] and the Royal Society's 2009 report, *Geoengineering the climate* [2]. There is a strong consensus that the governance questions raised by scientific research and technological developments in this area are profound [2]. In 2010, the United Nations Convention on Biological Diversity responded to mounting concerns about the implications of climate engineering by introducing a de facto moratorium, while recognizing the complexities of defining it (decision X/33 and IX/16 C). In 2013, the London Convention on the Prevention of Marine Pollution by Dumping and Other Matter and its Protocol also introduced rules prohibiting ocean climate engineering in the absence of a permit [3].

The emergence of intergovernmental debates around climate engineering also raises underlying questions about research governance. Scientists [4], ethicists [5] and others [2] have drawn attention to the possibility that permitting research on climate engineering could itself be a step onto a slippery slope, making development and eventual deployment of a technology more likely. We can say relatively little at this stage about the technological details of any future climate engineering system. But historical analogues would suggest the possibility for various forms of technological ‘lock in’ [6], ‘path dependen’ [7], ‘entrenchment’ [8] or ‘entrapment’ [9], whereby social and technological choices are constrained by pre-existing technological commitments, norms or standards. One does not have to adopt a position of technological determinism to acknowledge that technologies may develop a momentum [10] that proves hard to govern. In the absence of an established governance framework, the practices of scientific research [11] and intellectual property [12] tend to shape the field and set trajectories for future development. As such, it is important to render visible the emerging patterns of research and patenting.

Recent decades have seen growing attention among social scientists to the possibilities of anticipatory governance [13], constructive [14] and real-time [15] technology assessment, upstream engagement [16,17], value-sensitive design [18] and responsible innovation [19]. These approaches try to broaden the discussion about the implications and directions of technology at a point at which they might still be governed in the public interest. Nevertheless, we still face the dilemma of control identified by Collingridge [8]: governance is possible at an early stage, but at this point the uncertainties are so substantial that we lack an evidence base from which to govern. By the time we are aware of the effects of particular technologies, the range of governance options may have narrowed. To address these problems, the concept of anticipatory governance is increasingly being applied to emerging technologies and climate change adaptation. Anticipatory governance demands evidence-based foresight, flexible adaptation strategies and monitoring capacity as a basis for action in addressing new and emerging technologies and complex governance challenges such as climate change adaptation [20,21,22].

In this article, we seek to contribute to the evidence base for anticipatory governance. We use scientometric and analytics-based approaches to the analysis of the existing scientific and patent literature on climate engineering, with a particular focus on solar radiation management (SRM) techniques. In the process, we extend and update existing research involving bibliometric approaches to the topic. We argue for methodological transparency in defining the subject matter, and advocate a modular approach to baselining and future exploration of climate engineering inside the scientific and patent literature to facilitate monitoring. By advancing capacity to capture the evidence base, we can also contribute to opening up a well-informed democratic debate on the desirability of climate engineering and appropriate responses to climate engineering proposals such as SRM [23].

## 2. Bounding the scientific and patent literature on climate engineering

Defining the boundaries of climate engineering presents particular challenges for a number of reasons: it includes a highly diverse set of proposals using old and new technologies, bound together only by the intention to use them to counteract climate change. It is gaining a great deal of attention in the policy and public realms, which might lead some researchers to attach the label to their own existing work, and others to avoid it, and it is also still rapidly evolving. Our initial approach was to use the accepted ‘canonical’ definitions of climate engineering as a starting point, and to build on earlier work, notably that of Belter & Seidel [24]. In particular, following work in nanotechnology, our aim was to design a simplified search strategy for interrogating literature databases that could provide an easy way to reproduce a common baseline while providing a modular approach to the future exploration of climate engineering [25]. At the same time, we aimed to extend existing research on climate engineering into the patent system by testing approaches to retrieving patent documents of potential relevance to the area.

We initially conducted searches using the topic field in the Web of Science core collection with the query (‘climate engineering’ or ‘geoengineering’ or ‘geo-engineering’ or ‘engineering climate’). The results were then imported into VantagePoint analytics and natural language processing software for detailed analysis. In a series of iterative steps using keywords and phrases from Web of Science metadata, further exploratory searches were performed to test data capture and adjust for noise. The final Boolean search query consists of six modules, connected by ‘OR’ operators, as follows:

(1) TS = (geoengineer* OR ‘climate engineer*’ OR geo-engineer* OR ‘negative emissions’)
(2) (‘ocean iron fertili*ation’ OR ‘ocean fertili*ation’ OR ‘fertili*ation of the ocean’
(3) (‘direct air capture’ OR ‘capture from ambient air’)
(4) (‘solar radiation management’ OR ‘albedo modification’ OR ‘albedo enhancement’)
(5) (‘carbon dioxide removal’ AND climate)
(6) (‘carbon sequestration’ AND soil AND mitigat* AND climate)

The objective of these modules is to capture the universe of publications on climate engineering while limiting the signal-to-noise ratio to a minimum of 1:1. The wild card character (*) captures suffixes (e.g. -ing) and spelling variations (notably ‘fertilisation’ or ‘fertilization’). In addition to module 1, which uses the main synonyms for climate engineering, we tested further modules in order to capture specific sets of articles which are explicitly about specific climate engineering techniques as defined by the 2009 Royal Society report, but did not use the terms of module 1. Modules were only included in the final search query if they added a significant number of new records and had a signal-to-noise ratio better than 2:1. The final search query returned 1341 documents to the end of 2013 and false positives were removed through a manual cleaning process. The decision was taken to exclude documents which made only passing reference to climate engineering, which focused on affecting a regional rather than global climate, or which used CDR techniques to offset particular local emissions, but to include documents which fitted standard definitions of climate engineering or geoengineering even though they did not actually use those terms. This process resulted in a final cleaned dataset of 825 records.

The basic modules presented above can be expanded and refined as the field of climate engineering develops. This approach also allows the construction of specialist queries to identify themes of interest such as SRM. To achieve this, we manually reviewed and classified the records into three broad categories: SRM, carbon dioxide removal (CDR) and ‘general geoengineering’ (where both classes of technique are discussed).

VantagePoint permits access to the Combined Keywords and Phrases from the Titles, Abstracts, Author Keywords and cited titles (Keywords Plus) in Web of Science data. A total of 21 675 terms were available for analysis to identify the dominant terms used by authors for each theme (electronic supplementary material). A total of 5960 terms were available for the SRM dataset of 193 publications. Using word stemming, we were able to identify the top words and phrases that capture the majority of SRM publications. Our analysis revealed that SRM publications are heavily loaded onto geoengineering/geo-engineering with 172 records (89%) using this term and climate engineering accounting for 24 records (12%). Phrases such as climate change (86 records), SRM (47) and albedo enhancement (34) also featured prominently in the data but did not dominate the landscape (electronic supplementary material). Individual terms were dominated by aerosols (100), stratosphere/stratospheric (86), atmosphere (76) and albedo (60). Based on this information, we constructed a query that will capture the majority of the literature on SRM while limiting noise. Thus, the following query in Web of Science captures 173 (89.6%) of our 193 SRM records.

(geoengineer* or ‘climate engineer*’ or geo-engineer* or ‘solar radiation management’ or ‘albedo enhancement’ or ‘albedo modification’) AND (solar or radiati* or albedo or stratospher* or aerosol* or atmospher*)

This consists of a modified version of module 1 that excludes negative emissions and brings SRM and albedo enhancement/modification to the fore. The second AND clause searches within the overarching geoengineering/climate engineering results for terms directly related to SRM. The remaining 20 publications are dominated by news items, editorial material and letters (15 records) that lack an abstract. This prevented efforts to expand capture beyond 90% for the full SRM data. However, this approach captured 152 (98%) of the 155 journal articles in our SRM data.

Patent research was then conducted using the commercial Thomson Innovation patent database. Research was initiated using the simple search string highlighted above, focusing on searches at the European Patent Office, the United States Patent and Trademark Office and the international Patent Cooperation Treaty in the period to the end of 2013. The research was subsequently expanded to a working version of the six-module Web of Science query and the number of jurisdictions expanded. Patent documents were manually reviewed and classified by technology area and according to whether they were directly or indirectly relevant to climate engineering. In the final stage, citations of earlier patent documents (cited) and forward citations (citing) were retrieved, manually reviewed and classified. The resulting working patent landscape was combined with the Web of Science dataset in VantagePoint. Authors who are also inventors were mapped using co-author as co-inventor, author affiliation and co-applicant as the main match criteria. Direct subject-matter matches between the scientific literature and patent documents were used in a small number of cases. Patent data were counted based on first filings (patent families) and global follow-on applications (family members) using the International Patent Documentation Centre (INPADOC) system. Data were visualized in Tableau analytics software and network mapping was performed using the open-source Gephi package using the Fruchterman–Reingold algorithm and partitioned using the Modularity Class algorithm [26]. Raw data from the literature and patent searches are provided in the electronic supplementary material. We now turn to data on climate engineering in Web of Science.

## 3. Scientific publishing on climate engineering

### (a) Trends in publications

**Figure 1.**
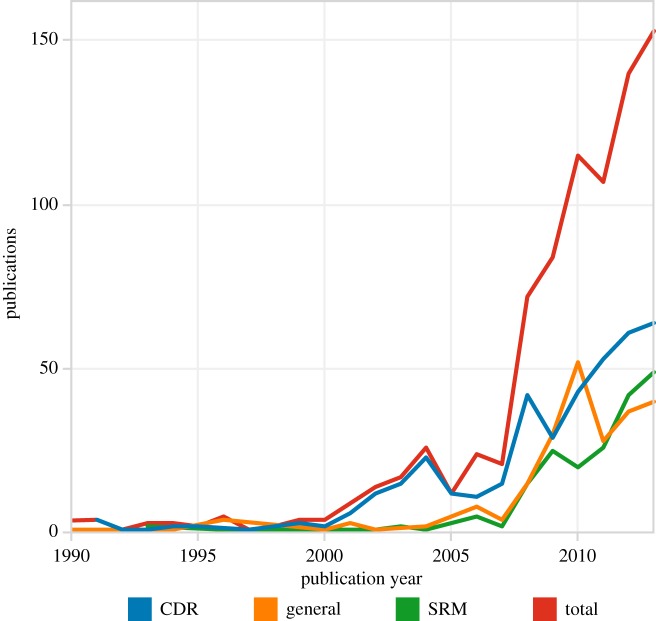
Main trends in scientific publications (Web of Science).

In total, we identified 825 publications consisting of journal articles, review items, news items, conference proceedings and books in the period between 1971 and 2013. Figure 1 displays publication trends by thematic types for SRM, CDR and ‘general geoengineering’. Our dataset was dominated by publications on CDR (404 records) followed by general geoengineering (228) and then SRM (193).

The idea of climate engineering has a long history [27,28]. Our data suggest that publications in the open scientific literature regarding large-scale interventions in climate can, at the very least, be traced back to the little known work of Lamb in a 1971 article in Earth Science Reviews on climate engineering schemes to meet a climatic emergency [29]. A few years later in 1977 Marchetti published an article on ‘Geo-engineering and CO_2_ problem’, the first article in our data to use this phrase to mean climate engineering [30]. A series of spikes are observable in figure 1 from the 1990s onwards. A first significant spike occurs in 1996 with the publication in *Climatic Change* of a selection of papers from the symposium organized at the 1994 annual meeting of the American Association for the Advancement of Science on whether humanity ‘could’ or ‘should’ engineer the Earth's climate (http://link.springer.com/journal/10584/33/3/page/1).

We see a small rise in publications to a peak in 2004. At this point, the literature begins to address topics such as soil carbon sequestration and ocean fertilization, with articles reporting on two 2002 ocean iron fertilization experiments, SOFeX in the southern ocean and SERIES in the Gulf of Alaska, and special issues on soil carbon sequestration in *Climatic Change* and *Journal of Arid Environments*. As noted by the 2009 Royal Society report, the extent to which soil carbon sequestration should be considered to be climate engineering (rather than mitigation) is open to question, but we include it in our working definition. While publications in 2004 are dominated by carbon sequestration in soils and oceans, we also observe a discussion on climate engineering and space solar power that involves a means of interacting with thunderstorms to prevent tornadoes [31]. The publication of Crutzen's classic 2006 article on SRM [1] is followed by a jump in the number of climate engineering publications in our dataset from 21 in 2007 to 72 in 2008. Interestingly, this surge is predominantly made up of articles on CDR. Top-cited publications in 2008 include a review of carbon dioxide separation and capture [32], greenhouse gas mitigation in agriculture [33], and abiotic and biotic technologies for carbon sequestration [34].

Discussion of SRM was limited to 16 publications between 1990 and 2007, but then accelerated. In our data, scientific publishing in this area was initiated by Penner in 1993 [35] with a proposal for a ‘low cost/no regrets approach’ to global warming by increasing the Earth's albedo by scattering small particles. Other highly cited publications can usefully be picked out from the subsequent years. A 2006 article by Wigley [36] proposed a combined mitigation/geoengineering approach using sulfate aerosol injection and attracted significant attention. SRM publications surged after 2007: 15 articles were published in 2008 alone, notably including Robock *et al.* [37] on ‘Regional climate responses with tropical and Arctic SO_2_ injections’ and Tilmes *et al.* [38] on the sensitivity of polar ozone depletion to geoengineering schemes. Influential SRM work in 2009 included Rasch *et al.* [39] on the influence of cloud seeding on sea ice and nonlinear climatic responses. In 2010, research by Ricke *et al.* [40] focused on critical assessment of regional climate responses to SRM and suggested that regional diversity in response rates could make it difficult to achieve consensus, while in *Science* Robock *et al.* [41] considered the potential pros and cons of SRM through aerosol injection. Significant publications in 2011 include work by Kravitz *et al.* [42] proposing a set of standard-forcing experiments tested against a range of climate models to evaluate stratospheric geoengineering using sulfate aerosols. An emphasis on sea spray climate engineering emerges in 2012 in work by Partanen *et al.* [43] and Alterskjaer *et al.* [44]. In 2013, Bony *et al.* [45] used simulations to examine the effect of carbon dioxide on the hydrological cycle focusing on tropical circulation and precipitation and concluded that climate engineering approaches that do not remove carbon dioxide would not fully mitigate precipitation changes in the tropics, and Parson & Keith [46] argued in *Science* for a need to ‘break the deadlock’ in geoengineering research on the grounds that such approaches may be needed in future.

Publishing trends on SRM need to be placed in the context of wider discussions of climate engineering. The overall upsurge of publications on climate engineering in 2008 coincided with increasing NGO and intergovernmental policy attention to the topic. In May 2008, the 9th Conference of the Parties to the United Nations Convention on Biological Diversity established a de facto moratorium on ocean fertilization by requesting that Parties ensure that ocean fertilization did not take place until there is an adequate scientific basis for justifying this decision (decision IX/16 C). This decision responded to concerns raised by NGOs led by the ETC Group, and is reflected in news items in *Science*, *Nature* and *New Scientist* during this period. In October of the same year, the London Convention agreed to set scientific guidelines for proposed ocean fertilization experiments (Resolution LC-LP.1(2008)). In the intervening period until the next peak in 2010, the 2009 LOHAFEX (Indian and German Iron Fertilization Experiment) in the South West Atlantic Ocean failed to sequester carbon and in 2009 the Royal Society published its report *Geoengineering the Climate: Science, Governance and Uncertainty* [2,47].

By 2010, the top-cited literature being published in the wider climate engineering field focused on issues such as carbon sequestration in grasslands [48] and indigenous agroforestry [49], but also reported the results of modelling regional climate responses to SRM [40]. The politics of climate engineering also received significant attention in *Science* [50], and the March 2010 Asilomar International Conference on Climate Intervention Technologies is reflected in news reports [51]. But in this same year, COP10 of the Convention on Biological Diversity decided, while recognizing issues around definitions of climate engineering, to extend its earlier decision beyond ocean fertilization to climate engineering as a whole (decision X/33). News items in this period are dominated by titles such as ‘Geoengineering faces ban’ [52] and ‘Geoengineers get the fear’ [53].

Although both SRM and CDR enjoyed a continuous rise in publications per year from 2010 to 2013, in 2011 a drop in the number of articles discussing geoengineering in general meant that the overall number of publications in our data dropped from 115 in 2010 to 107 in 2011. But this was followed by a notable upswing in 2012 to 140 publications with 101 articles. Highly cited publications from 2012 focus on issues such as soil organic matter dynamics [54], the 2004 EIFEX iron-enrichment experiment in the Southern Ocean [55], and a new experiment in the north-eastern pacific that prompted controversy over whether it breached the UN CBD and London Convention moratoria [56]. Both 2012 and 2013 show a surge in publications actually using the term ‘geoengineering’, and in reports of studies using computer simulations of climate engineering interventions. Data for 2013 are likely to be partial due to delays in database acquisition; however, an overall increase in publications to 153 records is observed. SRM publications in 2013 totalled 49 and were boosted by a special issue of *Climatic Change* on ‘Geoengineering Research and its Limitations’ that included articles on solar geoengineering [57], public engagement [58] and economics [59].

This brief overview points to issues in the emergence and evolution of climate engineering debates that merit further exploration. We now turn to the ‘who’, ‘what’ and ‘where’ of climate engineering research.

### (b) Key actors

We identified approximately 667 organizations from 67 countries in the overall data for climate engineering. Approximately 148 organizations from 26 countries are involved in research publications that focus solely on SRM (as opposed to publications on CDR and those on ‘general geoengineering’ which discuss both SRM and CDR). The US National Centre for Atmospheric Research is the most published organization in climate engineering research as a whole with 30 publications; it also leads on SRM with 21 publications, including influential work on aerosol injection [36]. Other top ranking organizations on SRM include the Max Planck Institute and Rutgers State University with 17 and 16 publications respectively, including highly cited work on the role of particle size in geoengineering using sulfate aerosols [60], and regional climate responses to geoengineering [37]. The UK Met Office ranks fourth with 13 publications, including highly cited work on the climate impacts of geoengineering marine stratocumulus clouds [61]. The Carnegie Institution for Science's Department of Global Ecology at Stanford ranks fourth with 12 publications [62] along with the University of Leeds [63]. A finer grained analysis is provided through examination of author networks.

### (c) Author networks

**Figure 2.**
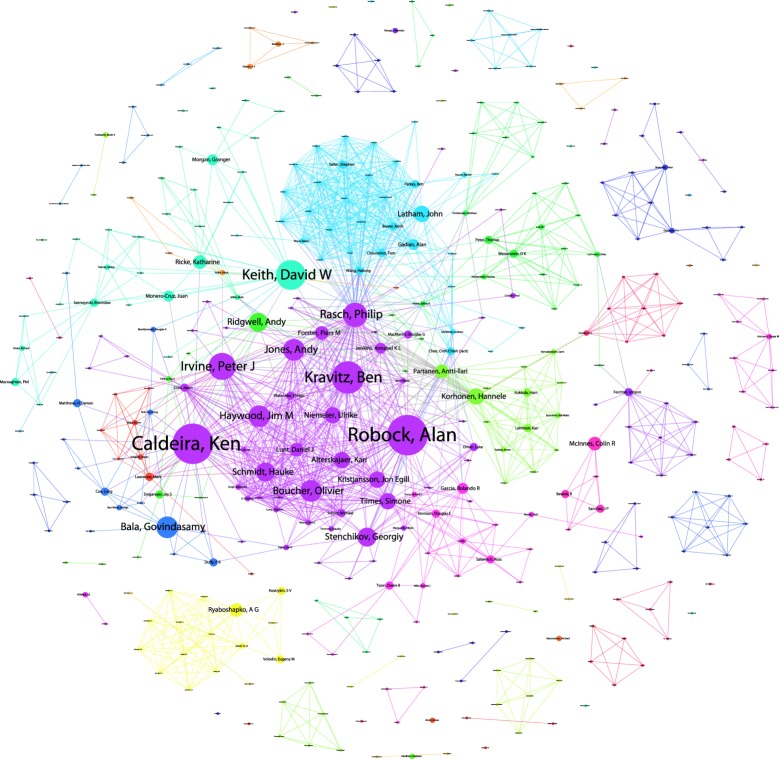
SRM Co-author network.

Following data cleaning, we identified approximately 1961 authors of publications in our Web of Science data. The majority of authors (1343) are publishing on CDR, with 401 authors for SRM and 325 authors for general geoengineering. Figure 2 displays a network map of co-authors of SRM-only research, with colours denoting communities based on the strength of co-authorship relations. Note that links (edges) in the network layout may transect unrelated nodes. In total, we identified 81 communities (including single-author isolates). The top ranking author, based on the number of publications, is Ken Caldeira, beginning with research with Govindasamy addressing radiative forcing [64], followed by influential work on climate-carbon simulations of geoengineering [62] and culminating, in our data, with consideration of the trade-offs in SRM arising from regional variations in its effects and equity considerations [65]. Work by Robock in the same cluster, focusing on Regional climate responses to SO_2_ injections, argues against Arctic and tropical geoengineering because of impacts on precipitation and food supply in Asia and Africa [37] and, with Kravitz, has more recently addressed sea spray geoengineering [66].

The networks of researchers working on climate engineering, including those working on SRM, comprise both senior researchers who have turned their attention to climate engineering, and junior researchers, usually working with senior colleagues, who would more obviously define themselves as ‘climate engineering researchers’. A focus on networks of researchers and organizations is a logical focus of governance measures, as are the funding organizations supporting this work, to which we now turn.

### (d) Funding networks

**Figure 3.**
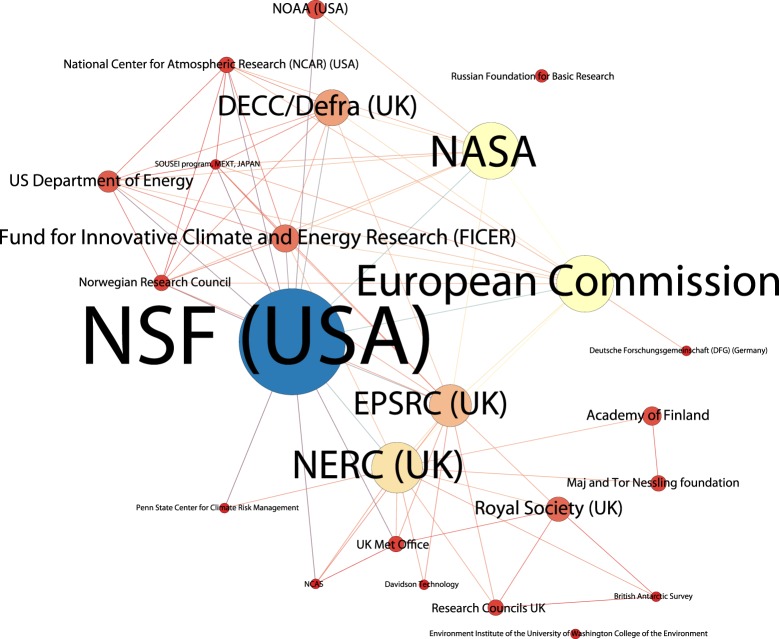
SRM funding network 2008–2013.

Information on funding bodies is only available for 34% of our data from 2008 onwards and requires extensive cleaning. However, information on the approximately 319 organizations listed in the funding acknowledgements provides useful insights into the underlying, and commonly hidden, networks of funding for research. Based on the available publication data, climate engineering research funding is dominated by the US National Science Foundation (NSF), the UK Natural Environment Research Council (NERC), the European Commission, the US Department of Energy and NASA with the National Natural Science Foundation of China appearing seventh in the rankings. Figure 3 displays the network of organizations funding SRM research acknowledged in two or more publications. (Note that the size of nodes indicates number of publications, not amount of funding.) This reveals a tighter concentration around the NSF, the European Commission and NASA. The scientific literature for climate engineering in general, but also that on SRM in particular, is dominated by research from public funding organizations. However, research is also being supported by non-governmental organizations such as the Fund for Innovative Climate and Energy Research (FICER) [66–68], which is funded from a gift by Bill Gates to the University of Calgary, and the Maj and Tor Nessling Foundation for research on sea spray geoengineering and marine cloud albedo [43,69]. We would emphasize that decisions on collaborations are typically made by individual researchers and reporting of collaborations may be limited. These networks are hidden from research funding organizations and hidden, beyond immediate collaborations, from researchers themselves. There has been relatively little strategic, directed research into climate engineering. It will be important for ongoing governance debates to keep watch on who is funding what, whether explicitly as top-down research on climate engineering or in emergent, bottom-up and often unnoticed networks of collaboration.

Research funders in the UK and elsewhere increasingly emphasize the pursuit of commercial innovation and spin offs from research. Patents provide a primary indicator of research and development directed towards potential commercialization but have received limited attention in the existing literature.

## 4. Patent activity on climate engineering

### (a) Trends in patents

We initiated the patent research using the Thomson Innovation whole-text patent database focusing on patent documents from the United States, the European Patent Office and the Patent Cooperation Treaty. Because of the unpredictable nature of the use of terms in patent data, we initially used the simple search terms ‘climate engineering’ or ‘geoengineering’ or ‘geo-engineering’ or ‘engineering climate’. The research was later expanded to use the six-module search term deployed in the literature research, and to include a wider range of jurisdictions. In particular, we reviewed all historic backward- and forward-citing patent documents from the keyword set to enhance data coverage.

We initially identified 101 raw results and it was immediately apparent that the search terms generated unexpected noise (e.g. geological engineering) and that the records were diffuse. We approached the data by allocating records to a ‘keep, review or exclude’ list in VantagePoint and progressively classified the records by the type of technology involved.

We identified 143 patent families (first filings) linked to 910 family members worldwide originating from 12 countries and published in 39 countries between 1971 and 2013. Table 1 provides a breakdown of the top categories of patent documents relating to climate engineering. Patent documents were classified as ‘direct’ if they made an explicit claim for use in climate engineering or to counteract global warming or produce planetary cooling through direct, large-scale interventions in climate. Patent documents were classified as ‘indirect’ where they described a well-known climate engineering technique, as classified by the 2009 Royal Society report, but did not make explicit reference to climate engineering. These include stratospheric aerosols, space-based reflectors and ocean fertilization and upwelling. The category ‘other’ refers to techniques that have not previously been well described but that could fall within the categories used by the Royal Society. The documents were subsequently categorized more generally into ‘CDR’, ‘SRM’ or ‘other’ as a mixed category. Note that patent documents may fall into more than one category.

**Table 1.** Climate engineering patent classification scheme.

theme	category	families	family members
CDR	direct air (indirect)	42	348
	direct air capture	4	56
	ocean fertilization (indirect)	9	129
	ocean fertilization	3	61
	ocean fertilization CO_2_ capture	12	130
	ocean up/downwelling	2	7
	ocean upwelling (indirect)	29	96
	ocean upwelling CO_2_ capture	5	20
	*sub-total*	96	683
other	other	19	98
	other (indirect)	6	25
	*sub-total*	25	123
SRM	cloud brightening	1	1
	space based	7	13
	space based (indirect)	2	4
	stratospheric aerosols (indirect)	2	4
	stratospheric aerosols	9	74
	surface albedo	7	59
	surface albedo (indirect)	1	18
	*sub-total*	28	170
total		143	910

Table 1 makes clear that patent activity that is indirectly related to climate engineering is a major component of the data with 91 indirect and 52 direct families. This disparity reflects the problem that patent claims are often deliberately constructed in a broad way by applicants to capture the maximum range of possible uses of a claimed invention. For example, patents for ocean fertilization make claims about increasing fish stocks as well as climate engineering. In selecting the documents, we sought to reflect the variety of definitions that could be used for climate engineering.

**Figure 4.**
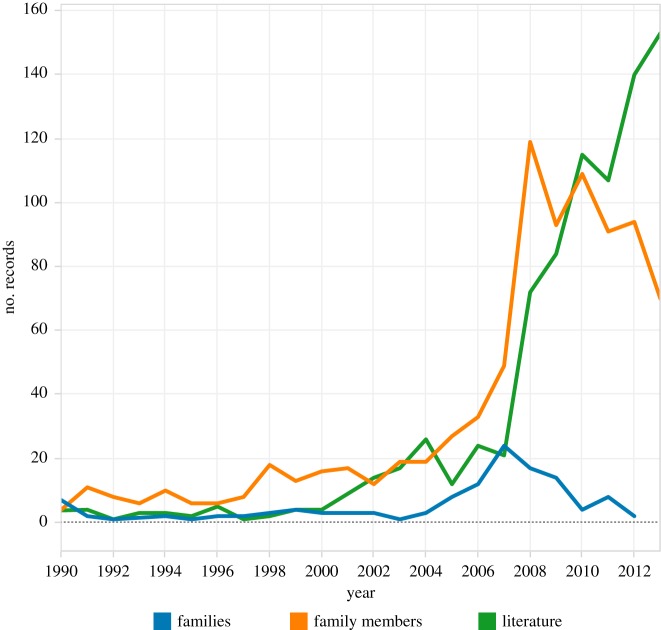
Patent trends and literature trends.

Figure 4 displays global trends in first filings and family members in climate engineering as a whole, compared with trends in scientific publications. In interpreting figure 4, we can observe that the rate of first filings or ‘families’ of patent applications (where an application is counted only once, so is more comparable with a scientific publication) is much lower than that of the scientific literature. Furthermore, patent filings peak in 2007, from which point we observe a declining trend followed by a low-level recovery in 2011. As we move towards 2012–2013, the availability of patent data rapidly declines; however, within the limitations of our search criteria it is reasonable to make the general observation that patent activity for climate engineering (direct or indirect) is presently at a very low level. This is confirmed in figure 4 by trends in global follow-on filings of patent applications (‘family members’), where documents are republished in multiple countries. Data for family members display a spike from 2008 to 2010 as first filings are republished and the underlying general backlog of pending patent applications is processed by patent offices. However, the rapid decline in family member trends between 2012 and 2013 will reflect a lack of data availability in patent databases for recent years.

Table 1 also reveals that CDR dominates the patent data with 96 filings and 683 family members worldwide. SRM displays very limited activity with 28 filings and 170 family members worldwide. The earliest filing related to SRM can be traced to 1967 from Westinghouse Electric Corp in a filing on a ‘System and Method for Irradiation of Planet Surface Areas’ (US3564253A) that focused on irradiating areas of the planet for illumination, heating or weather control using one or more satellites. Another early example from 1989 is a filing by a Japanese inventor for a protective apparatus placed in space that would project a solar ray shadow to a specific area of the planet and contribute to addressing global warming (WO1990010378A1). This is followed in 1991 by the publication of a US patent grant awarded to Hughes Aircraft Co for ‘Stratospheric Welsbach Seeding for Reduction of Global Warming’ (US5003186A). The Hughes Aircraft Co. filing describes a method involving dispersing tiny particles of material with high reflectivity for radiation in the visible or infrared spectrum and low emissivity in the near infrared spectrum to convert infrared heat energy for radiation into space. This patent application has been cited by a number of later applicants. A 2007 filing by Leslie Field focuses on a system for environmental modification with climate control materials and coverings that can be used to modify local albedo and evaporation rates in the planetary ecosystem (WO2009048627A2).

A 2011 filing by the Korea Aerospace Research Institute provides a ‘Method for controlling Land Surface Temperature using Stratospheric Airships and Reflector’ (WO2013077557A1). A 2011 application from a US inventor focuses on a system for ‘releasing or capturing disbursements for the atmosphere by means of an aircraft’ that involves a system of hoses for ozone replenishment, protection against UV light and storm degradation (US20110284690A1). A recent application from four US individuals, including Armand Neukermans linked to the Fund for Innovative Climate and Energy Research (FICER), provides for salt water systems for cloud brightening using nanoparticles and is presently awaiting examination (WO2013086542A1). We now focus on the characteristics of applicants for patent rights in climate engineering.

### (b) Patent applicants

Table 2 displays the top patent applicants across our dataset based on counts of patent families as a measure of claims to invention and counts of patent family members as an indicator of demand for patent rights by the applicants in multiple countries. Levels of individual filings are low, with the clear leader being Global Research Technologies (co-founded by Klaus Lackner and Allen Wright of Columbia University), which was later renamed Kilimanjaro Energy. However, one of the most striking features of applicants is the presence of two intellectual-property-only companies Searete LLC and Invention Sci Fund. In the case of SRM, these companies have received a US patent grant for a ‘High altitude structure for expelling a fluid stream through an annular space’ (US8166710B2) that envisages uses to influence global warming or cooling. Both companies are reportedly subsidiaries of Intellectual Ventures Inc. (IV), an intellectual property company that acquires and licenses patents and patent applications and has developed in-house research capacity as Intellectual Ventures Lab. Intellectual-property-only companies have been associated in wider public discourse with the phenomenon of ‘patent trolls’, notably in the field of computer software patents [70,71]. The presence of IP-only companies could suggest strategic positioning to capitalize on potential developments in climate engineering, particularly if broad patent grants have been awarded on fundamental aspects of emerging climate engineering technologies. This merits further investigation beyond the present research.

**Table 2.** Top patent applicants.

patent applicants	families	family members
Global Res Technologies LLC	13	180
Kilimanjaro Energy Inc.	8	159
Greensea Venture Inc.	1	46
Cantrell Winsness Technologies LLC	1	44
GS Cleantech Corp	1	44
Invention Sci Fund I LLC	2	39
Searete LLC	2	39
Liquid Robotics Inc.	1	34
Univ Columbia New York	3	33
Univ Leland Stanford Junior	1	32
Davidson Technology Ltd	1	21
Mitsubishi Jukogyo KK	3	20
Mitsubishi Heavy Ind. Co. Ltd	2	19
Alcan Technology and Management AG	1	18
Alsuisse Technology and Management AG	1	18
Alusuisse-Lonza Services Ltd	1	18
Du Pont De Nemours and Co E I	1	17
Geolink UK Ltd	1	17
Harvard College	1	17
Penn State Res Found	1	17
Sondex AS	1	17
Geophysical Eng Co.	2	14
Univ. Princeton	1	13
Univ. Southern California	1	13

Patent applicants in table 2 are ranked in terms of the number of family members worldwide as an indicator of their efforts to secure patent rights in more than one jurisdiction. The most important patent documents in applicants’ portfolios are revealed through patent citation scores—that is, the number of times they are cited by later patent applicants. In the case of Global Research Technologies, the portfolio mainly focuses on air capture for CO_2_ with the highest cited document being an application for a ‘Method and apparatus for extracting carbon dioxide from air’ with 83 citations (US20080087165A1). However, when looking across the dataset the single most important document is a 1987 patent grant to Air Products & Chemicals Inc. for ‘Removal of Water and Carbon Dioxide from Atmospheric Air’ with 118 citations that describes a system of adsorptive removal of moisture and CO_2_ from atmospheric air by passing air through consecutive beds of adsorbent (US4711645A).

In practice, patent documents directed towards capture of CO_2_ dominate the top-cited documents across the landscape and include approaches such as electrochemical methods to generate hydrogen and sequester carbon dioxide (US20080245672A1). For ocean fertilization, the most important patent is a method of increasing seafood production in the ocean and applying a fertilizer consisting of a nitrogen-fixing microorganism before harvesting the seafood resulting from the fertilizations (US5535701A). Here, ocean fertilization is primarily represented as a means to enhance seafood productivity. In the case of space-based SRM-related approaches, the most relevant documents are from Westinghouse (see above) followed by two grants to an individual relating to solar powered aeroplanes and a satellite weather modification system (US6045089A and US5984239A).

### (c) Inventors

**Figure 5.**
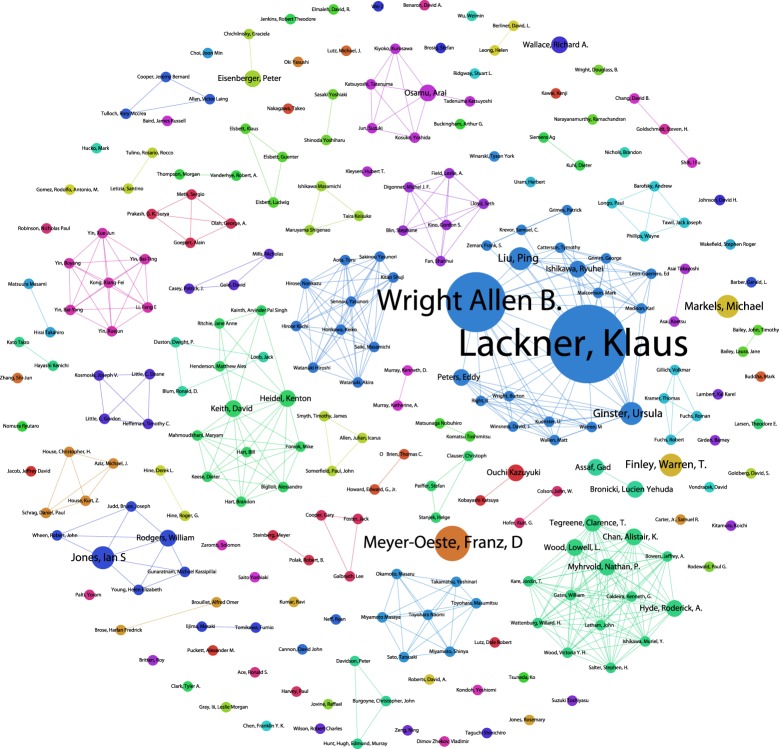
Climate engineering inventor network.

In total, we identified a very small field of 244 inventors in the patent data for climate engineering. Of these, approximately 22 were also the authors of scientific articles in our Web of Science dataset. These authors also dominate the emerging patent landscape for climate engineering in terms of the number of filings and family members. It is important to note that our data is based on documents identified using keyword searches and may therefore not reflect the full portfolio for each individual. Figure 5 displays a network map for all inventors in the emerging patent landscape.

The work of Klaus Lackner consists of 12 filings focusing on air capture of CO_2_. Eight of these filings are shared with Allen Wright. Ursula Ginster has submitted three filings in collaboration with Allen Wright focusing on capture of CO_2_ (i.e. US20120304858A1). Ken Caldeira has filed for a method and apparatus for extracting and sequestering CO_2_ (US20010022952A1) along with a structure for capturing wave water (WO2010138195A1). Three filings from Michael Markels in the early 2000s include methods for increasing seafood production and fish catch in the ocean along with a US patent grant for a method of sequestering carbon dioxide with chelated iron awarded in 2002 (EP760597B1, WO2004045274A2 and US6440367B2). In terms of SRM activity, Franz Dietrich Oeste has filed three families directed towards tropospheric cooling (e.g. WO2003013698A2 and WO2010075856A2) while Peter Davidson *et al.* have filed for a delivery system for delivering sub-micrometre particles into the atmosphere (e.g. WO2011073650A1).

The existing data suggest that small networks of inventors associated with particular companies and a number of individual inventors are dipping a toe into the patent system. Many patents are highly speculative and appear unlikely to survive the examination process. We identified a total of 28 patent filings for SRM, suggesting very limited activity in this field. While patent activity appears to be minor it merits further research using an approach focusing on capturing activity by individual companies and inventors rather than relying on keyword searches. Furthermore, our existing research does not include Bioenergy with Carbon Capture and Storage (BECCS) approaches such as biochar that merit further exploration. The sizes of existing patent families and citation scores in our data do not suggest that any inventor or company has laid claim to the climate engineering equivalent of recombinant DNA technology, Taq DNA polymerase or green fluorescent protein in the field of biotechnology. As noted above, the presence of an IP-only company in the data could suggest strategic positioning in the event that climate engineering technologies take off in the future. The emergence of climate engineering is unlikely to mirror that of biotechnology, because the range of proposed technologies is so wide. Technologies for high-leverage proposals such as stratospheric particle injection or ocean iron fertilization are, if they are taken forward, likely to be a form of ‘bricolage’, combining and repurposing technologies from other domains. Furthermore, the claims of patent documents that may be relevant for climate engineering seem to reflect scientific and political fashions over time; thus, recent patent documents make claims about global climate change while in previous decades, claims are more likely to relate to ozone layer protection or hurricane suppression. Researching patent documents may therefore provide a lens for only a small part of the technology picture, and one which is distorted by wider societal trends. However, we would still maintain that patent activity is an important governance consideration, as has been argued by others [12,72].

We now turn to discussion of the effectiveness of our approach to the monitoring of climate engineering scientific literature and patent data as a contribution to debates on democratic deliberation and governance.

## 5. Using bibliometrics to monitor the field

Scientometric approaches and an increasing shift to advanced large-scale text mining and analytics can contribute to illuminating the landscape of emerging areas of science and technology to inform debate and decision-making. However, the use of such approaches also creates challenges in terms of baselining and methodological transparency. In the first case, the challenge of generating baseline data reflects linguistic ambiguities in the subject matter (e.g. climate engineering or geoengineering; SRM or albedo modification) and disagreements about what should or should not be considered to fall within the subject area. The second problem relates to the variety of search criteria that researchers may use, the variety of data sources and the availability of raw data to permit follow-on research. In particular, opportunities for direct comparative analysis of data are rare in the social sciences.

**Figure 6.**
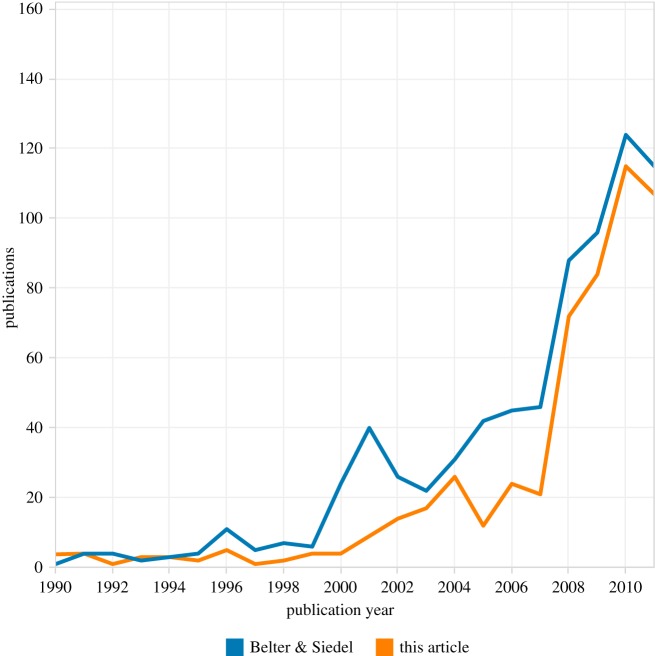
Comparing approaches to capturing the climate engineering literature.

As noted above, our research was informed by existing bibliometric work on climate engineering literature, notably the work of Belter & Seidel [24]. Our aim in the present work was to produce a simplified, modular approach to the search strategy used in previous research. At the same time, we sought to extend the analysis into the exploration of patent data. An important enabling feature of Belter and Seidel's work is that they made available the raw data in the form of ISI Unique identifiers. Figure 6 displays a direct comparison of Web of Science data in this article with previous work by Belter and Seidel for the period to the end of 2011.

Figure 6 demonstrates that the present research reveals a longer history of climate engineering research than is presented by Belter and Seidel. As we move into the late 1990s, their data also displays a marked spike around 2001 that is not reflected in the present research, while a marked trough is displayed in our research between 2004 and 2007 that is not observable in their study.

To investigate these differences, we compared the ISI unique identifiers for the corresponding periods to identify records in Belter and Seidel that do not appear in our work. Of the 35 records outside our dataset in 2001, 12 records were articles in a special issue of *Deep-Sea Research Part II* on the 1999 Southern Ocean Iron Release Experiment (SOIREE). The remaining variation for 2001 consisted of records addressing soil carbon sequestration and agriculture. Of the 114 publications between 2004 and 2007 that were not in our dataset, these were predominantly on ocean-related (36), soil-related (29) and agricultural (20) topics.

These extra records in Belter and Seidel's dataset could be explained in a number of ways. First, their search query used wild cards and the NEAR operator, which would mean, for example, that it will return not just records containing ‘ocean fertilization’ but also those with ‘fertilizing the Southern Ocean’; however, in practice this does not seem to have caused much of the variation. Second, Belter and Seidel included search terms on biochar and enhanced weathering; however, modules using these terms were tested by the current authors and found not to produce significant numbers of extra records. Third, they also used backward and forward citations and the WoS ‘related records’ facility to expand the list produced by their search query to include other relevant articles. This seems to have been the main reason for Belter and Seidel picking up records that we did not. However, we think that our search query could be adapted to capture the bulk of the ‘missing’ records.

On the other hand, the current study identified records before 1988 and captured 208 publications between 1991 and 2011 that were not in the earlier work; these were dominated by climate engineering and carbon sequestration (104), and ocean fertilization (28). Examining the respective search terms, this seems to be largely a result of our use of a more extensive WoS subscription, in terms of titles and the time period covered.

We argue that our approach to climate engineering bibliometrics has been found to be effective. Guided by an emphasis on transparency, repeatability and adaptability, our aims have been (i) to have a modular search query, which captures the ‘core’ literature using the term and its synonyms, literature on specific climate engineering techniques, and other basic scientific research that is being drawn into the climate engineering debates; (ii) to have a search query which captures the vast majority of relevant records but is relatively low on false positives that need manual cleaning; and (iii) to restrict the final dataset to those records returned by the search query. Added to this must be the general principles of making available the raw data, and the criteria used for manual cleaning.

Patent data present particular challenges, because search terms that are considered ‘clean’ in literature databases may produce unexpected surprises in patent databases. Furthermore, patent claims are often constructed in broad terms such that it can be difficult to determine whether a document is directly or indirectly relevant to climate engineering. Variations in patent database coverage and the availability of multiple legitimate ways of counting patent data can also produce problems in comparability.

Patent landscape analysis of the type presented in this article is typically performed using a combination of key terms and patent classification codes to confine the search to specific areas. One problem in the case of climate engineering is that there is no specific classification code for these emerging technologies to assist with targeting research. The Cooperative Patent Classification adopted by the European Patent Office and the United States Patent and Trademark Office includes a classification scheme for climate mitigation under classification code Y02. However, our data reveal that climate engineering is dispersed across areas of the classification such as agriculture, ocean energy, aircraft and space vehicles (electronic supplementary material). Growing interest in patent landscape analysis at the World Intellectual Property Organization and at the European Patent Office suggests the possibility of collaborations in future research to capture and explore climate engineering to inform policy debates [73].

## 6. Conclusion

This article has aimed to contribute to democratic deliberation on the governance of climate engineering in general, and SRM in particular, by seeking to make visible emerging patterns and structures in scientific research and patent activity. In doing so, we have deliberately sought to build on existing work with the aim of establishing a common baseline for monitoring climate engineering that allows for flexibility and clarity in defining climate engineering and also accommodating emerging developments through a modular approach. As the discourse of climate engineering alters, although there is no objective way to define its shifting boundary, existing modules can nevertheless be adjusted or new ones added in a transparent way in order better to capture the changing landscape [74,75]. Furthermore, we advocate transparency in making raw data available for comparative analysis by research teams to permit the stabilization of a consensus baseline over time. Finally, we have argued that greater clarity on patent activity for climate engineering could be achieved through cooperation with the World Intellectual Property Organization and the European Patent Office to facilitate the targeted identification of climate engineering activity under a variety of definitions.

Marking the edges of climate engineering as an issue, research domain or area of innovation is exceptionally challenging, given the profound scientific and definitional uncertainties. Nevertheless, we have argued that the bibliometric monitoring of research and patenting activity could constitute an important part of the anticipatory governance of climate engineering. It is valuable because of its capacity to make visible the often-hidden networks of collaboration, funding and problem-definition involved in emerging areas of science and technology, and to provide a transparent evidence base that can inform assessment and democratic deliberation.

## Supplementary Material

Supporting data tables

## Data accessibility

Raw data will be deposited in a recognized repository before publication of the article.
